# Ambulance Facilities and Telephone Efficiency

**Published:** 1921-02-19

**Authors:** Ralph B. Cannings

**Affiliations:** Secretary. City of London Maternity Hospital, City Road, London, E.C. 1


*To the Editor of* The Hospital.

### Ambulance Facilities and Telephone Efficiency.

*To the Editor of* The Hospital.

Sir,—Referring to the suggestion, quoted in your current issue, that the London County Council should provide a night ambulance service for lying-in cases, it may not be generally known that, as the result of representations made to the department responsible, the experimental service operating in four south-side boroughs was extended, as from January 1, to the Boroughs of Bethnal Green, Shoreditch, Finsbury, and Stepney. As, however, the ambulance has to be called by telephone, and telephone call-offices open at night seem to be almost impossible to find, the success (to the public) of the service would appear to depend upon a radical improvement of telephonic facilities during the night-time.—Yours truly,

Ralph B. Cannings, Secretary.

City of London Maternity Hospital,
City Road, London, E.C. 1,
February 14, 1921.

